# Profiling RNA G‐Quadruplexes *In vivo*

**DOI:** 10.1002/cpz1.70209

**Published:** 2025-09-19

**Authors:** Bibo Yang, Yiliang Ding, Yueying Zhang

**Affiliations:** ^1^ Department of Cell and Developmental Biology, John Innes Centre Norwich Research Park Norwich United Kingdom

**Keywords:** G‐quadruplex, RNA structure profiling, SHALiPE‐Seq

## Abstract

RNA G‐quadruplexes (RG4s) are specific and complex tertiary structures that form in guanine‐rich regions of RNA and can be detected *in vitro*. A recently developed transcriptome‐wide technique, SHALiPE‐Seq, enables the assessment of RG4 folding status within living cells in a quantitative manner. This method integrates chemical probing with high‐throughput sequencing. SHALiPE‐Seq relies on the property of 2‐methylnicotinic acid imidazolide (NAI), which preferentially modifies the last guanine in G‐tracts when RG4s are folded. To establish reference profiles, *in vitro* NAI modification patterns are generated under potassium ion (K⁺) conditions, which promote folding, and lithium ion (Li⁺) conditions, which maintain RG4s in an unfolded state. By comparing *in vivo* SHALiPE‐Seq profiles with these *in vitro* benchmarks, it becomes possible to identify and evaluate the formation of RG4s in living cells. Although this protocol has been applied to *Arabidopsis thaliana* and rice, SHALiPE‐Seq is broadly applicable to other systems and provides a valuable approach for investigating the *in vivo* dynamics of RG4s and their potential biological functions. © 2025 The Author(s). Current Protocols published by Wiley Periodicals LLC.

**Basic Protocol 1**: *In vitro* and *in vivo* NAI probing of RNA

**Basic Protocol 2**: Construction of the SHALiPE‐Seq libraries

**Basic Protocol 3**: Measurement of the RNA G‐quadruplex folding status based on SHALiPE‐Seq libraries

## Introduction

RNA G‐quadruplexes (RG4s) are tertiary structural motifs that form in guanine‐rich sequences through the stacking of two or more G‐quartets stabilized by Hoogsteen and Watson–Crick base pairing (Fay et al., 2017; Kwok & Merrick, 2017). RG4s have been implicated in various biological processes, such as polyadenylation (Beaudoin & Perreault, 2013), alternative splicing (Didiot et al., 2008), mRNA localization, transcript stability (Ishiguro et al., 2016; Zalfa et al., 2007), and translational regulation (Kwok et al., 2015; Lyu et al., 2019; Yang et al., 2020) across multiple species (Fleming et al., 2016; Kwok et al., 2015; Kwok et al., 2016a; Shao et al., 2020; Yang et al., 2020). In plants, RG4s were found to influence plant growth and development (Cho et al., 2018). An example is that RG4s can regulate vascular development. JULGI (JUL) is an important regulator of phloem differentiation (Cho et al., 2018). JUL binds RG4 structures in the 5′ UTRs of *SMXL4/SMXL5* mRNAs, thereby suppressing their translation and inhibiting phloem differentiation (Cho et al., 2018). Additionally, RG4s were also found to regulate root development by triggering liquid–liquid phase separation or function as RNA structure switches facilitating plant responses to environmental changes (Zhang et al., 2019; Yang et al., 2022). Most previous studies on RG4s have been performed under *in vitro* conditions. However, the folding behavior of RG4s within living cells remains less well understood. Given the complexity of the intracellular environment, RNA structures formed *in vivo* may differ significantly from those formed *in vitro* (Ding et al., 2014; Ganser et al., 2019; Rouskin et al., 2014). Therefore, characterizing RG4 formation *in vivo* is essential for elucidating its functional importance in cellular processes.

In the past decade, the integration of high‐throughput sequencing and *in vivo* RNA structure chemical probing has substantially advanced research on RNA structures *in vivo* (Ding et al., 2014). Chemical treatment with dimethyl sulfate (DMS) and selective 2′‐hydroxyl acylation, analyzed by primer extension (SHAPE) as representative types, can penetrate the cells and modify the single‐stranded regions of RNAs. After deep sequencing, the modification signals can be transformed into massive data, reflecting the single‐strandedness of every nucleotide of RNAs at the transcriptome level. This method has significantly advanced the study of RNA structures *in vivo*. For RG4s, SHAPE‐type chemicals preferentially modify each G‐tract's last G residue of RG4, which establishes a prerequisite for developing RG4‐specific structure profiling methods (Guo & Bartel, 2016; Kwok et al., 2016b; Yang et al., 2020). Additionally, since RG4 stability is modulated by cations, with potassium (K⁺) being more effective than lithium (Li⁺) (Bugaut et al., 2010), RNA treated *in vitro* with SHAPE under K⁺ and Li⁺ conditions can serve as benchmarks for folded and unfolded RG4 states, respectively. Based on these foundations, we developed SHALiPE (Selective 2′‐Hydroxyl Acylation with Lithium ion‐based Primer Extension)‐Seq to capture *in vivo* folded RG4 in whole transcriptomes.

SHALiPE‐Seq is the first transcriptome‐wide method that allows quantitative measurement of RG4 folding states *in vivo*. By integrating chemical probing with high‐throughput sequencing, this technique enables nucleotide‐resolution analysis of RG4 structures across tens of thousands of RNAs in a single experiment. SHALiPE‐Seq contains three different samples treated using NAI (2‐methylnicotinic acid imidazolide, a widely used SHAPE‐type chemical) under three conditions: *in vivo*, *in vitro* with K⁺, and *in vitro* with Li⁺. The *in vitro* samples under both ionic conditions are prepared alongside the *in vivo–treated* RNA for comparative analysis after deep sequencing. The chemical modifications introduced by NAI are detected from the sequencing reads. Following data acquisition, a computational pipeline is applied to quantitatively assess RG4 folding at the transcriptome level. SHALiPE‐Seq has been applied successfully to both *Arabidopsis thaliana* and rice, and *in vivo* folded RG4s were found to exist and function as an RNA stabilizer in plants (Yang et al., 2020; Yang et al., 2022).

**Figure 1:**
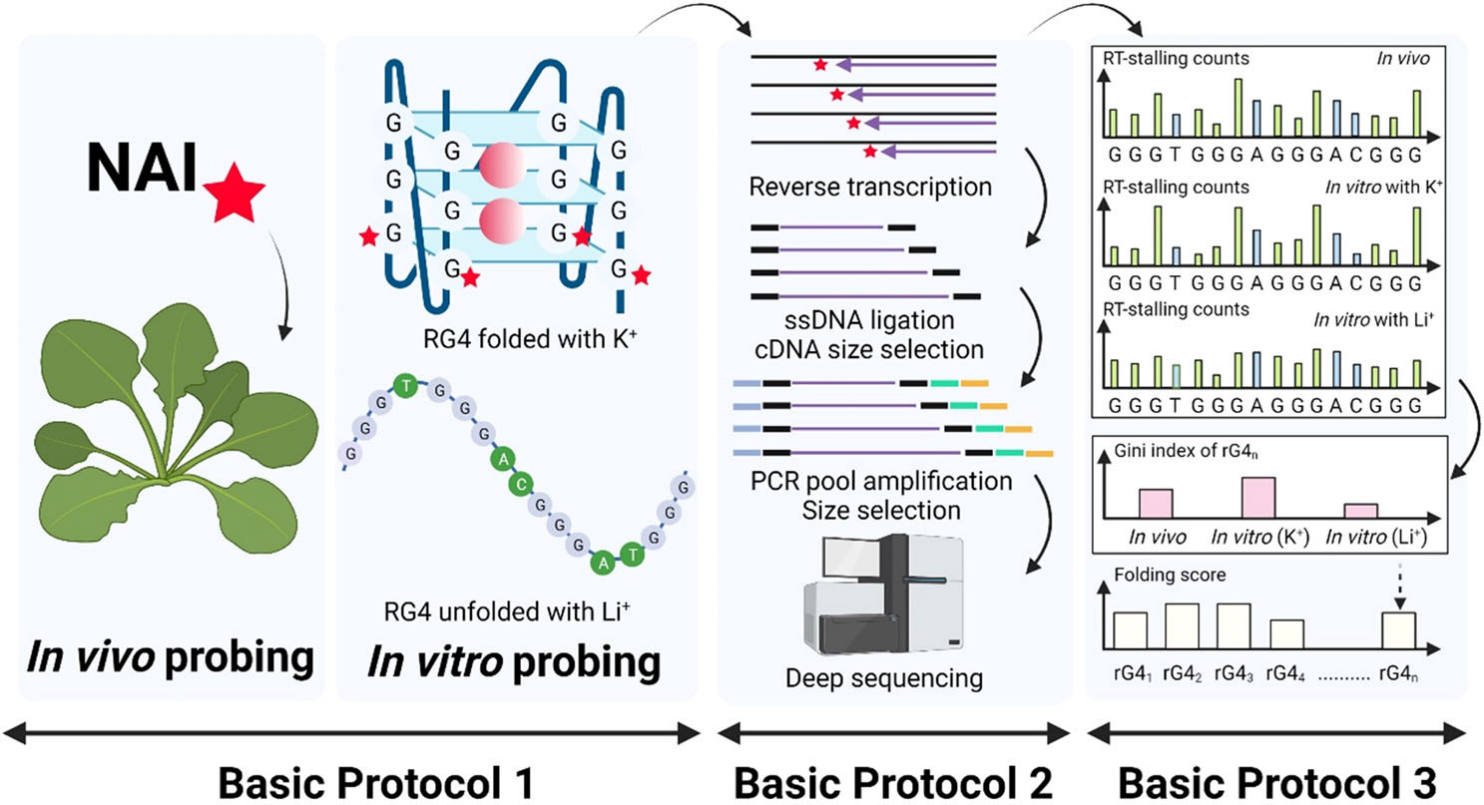
Overview of the SHALiPE‐Seq protocols described in this article.

This paper outlines a detailed SHALiPE‐Seq protocol using *Arabidopsis thaliana* as a model system. While demonstrated in plants, the method is broadly applicable to other organisms. An overview of the workflow is illustrated in Figure 1.

## Strategic Planning

We established and implemented SHALiPE‐Seq in *Arabidopsis thaliana*, a widely used model plant, because of the key role of cellular K⁺, the most abundant ion in regulating plant development and stress responses (Britto & Kronzucker, 2008; Wang et al., 2013). In our experiments, seeds of the *Arabidopsis thaliana* Columbia‐0 (Col‐0) ecotype were sourced from the Nottingham Arabidopsis Stock Centre (NASC). Before initiating the SHALiPE‐Seq experimental procedures, the plant materials underwent preparatory treatment.

1. Seeds were surface sterilized in 70% ethanol (20 min), rinsed three times with distilled water, sown in ½ MS medium with 1% (w/v) sucrose, stratified at 4°C in darkness for 3 days, and transferred to a 22°C growth chamber.

SHALiPE‐Seq is an NAI‐based chemical probing technique specifically designed for detecting RG4s, integrating selective 2′‐hydroxyl acylation with lithium ion–based primer extension (SHALiPE) and high‐throughput sequencing. The protocol begins with the synthesis of NAI and includes RNA probing both *in vitro*, under K⁺ and Li⁺ conditions, and *in vivo*. After library preparation and sequencing, the second phase involves computational analysis to quantitatively assess the folding states of RG4s across the transcriptome.

*NOTE*: All protocols involving animals must be reviewed and approved by the appropriate Animal Care and Use Committee and must follow regulations for the care and use of laboratory animals. Appropriate informed consent is necessary for obtaining and use of human study material.

## Basic Protocol 1 *IN VITRO* AND *IN VIVO* NAI PROBING OF RNA

This protocol describes the methodology of profiling the formation of RG4s *in vitro* and *in vivo*. The first part describes the synthesis of the NAI chemical that was first established in previous studies (Kwok et al., 2013; Spitale et al., 2013). The *in vivo* and *in vitro* NAI probing of RNAs is briefly described in our previous study (Yang et al., 2020). For both *in vitro* and *in vivo* NAI probing, at least two biological replicates for each condition should be prepared. In summary, two *in vivo* NAI probing libraries and four *in vitro* probing libraries generated in the presence of K^+^ or Li^+^ will be required for the SHALiPE‐Seq construction.

### Materials

2‐methylnicotinic acid (Sigma, 325228)
Dimethyl sulfoxide (DMSO, anhydrous) (Sigma, 276855)
1,1′‐carbonyldiimidazole (Sigma‐Aldrich, 21860)
5‐day‐old etiolated *Arabidopsis thaliana* Col‐0 seedlings
RNeasy Plant Mini Kit (Qiagen)
Ambion Poly(A) Purist MAG Kit (Thermo Fisher Scientific, AM1922)
Tris·HCl (pH 7.5) (Sigma‐Aldrich, T2319)
MgCl_2_ (Sigma‐Aldrich, 63020)
LiCl (Sigma‐Aldrich, L7026)
KCl (Sigma‐Aldrich, 60142)
Dithiothreitol (DTT) (Thermo Fisher Scientific, R0862)

Micro Bio‐Spin Columns Bio‐Gel P‐6 (Bio‐Rad Laboratories, 7326221)
Vortexing machine
Rotary Shaker (Eppendorf Thermo Mixer)
Refrigerator
Thermo Cycler for PCR
Eppendorf tubes

#### Synthesis of NAI

1 Weigh 137 mg (1 mmol) of 2‐methylnicotinic acid (2‐methylpyridine‐3‐carboxylic acid) and dissolve it in 500 µl of anhydrous dimethyl sulfoxide (DMSO) using a 1.7‐ml Eppendorf tube. Briefly vortex the mixture occasionally at room temperature to ensure complete dissolution.
2 In a separate 1.7‐ml Eppendorf tube, dissolve 162 mg (1 mmol) of 1,1′‐carbonyldiimidazole in 500 µl of anhydrous DMSO.
3 Gradually add the carbonyldiimidazole solution (from step 2) into the 2‐methylnicotinic acid solution (from step 1) over approximately 5 min, intermittently vortexing to mix thoroughly.
4 After combining, briefly vortex the final mixture again at room temperature to ensure proper reaction initiation.
  Occasionally loosen the tube cap while vortexing to allow any released gas to escape and continue until gas evolution ceases.
  The resulting solution from step 4 serves as a 1.0 M stock containing a 1:1 mixture of NAI and the byproduct imidazole.
  Store the stock at −80°C and keep it frozen when not in use. Before opening, allow it to thaw at room temperature.
  CAUTION: NAI is hazardous; appropriate personal protective equipment, such as gloves and safety glasses, should be worn during handling.

#### In vitro NAI probing of RNA

5 Extract total RNA from 5‐day‐old etiolated *Arabidopsis thaliana* Col‐0 seedlings using the RNeasy Plant Mini Kit.
6 Perform two rounds of poly(A) selection using the Poly(A) Purist Kit.
7 Mix 1 µg of the poly(A)‐selected RNA in a solution containing 50 mM Tris·HCl (pH 7.5); we use 0.5 mM MgCl_2_, 100 mM LiCl, or 100 mM KCl for the unfolded/folded G‐quadruplex benchmarker.
8 Heat the RNA mixture at 95°C for 90 s to denature secondary structures.
9 Chill the mixture on ice for 2 min to allow proper refolding.
10 Equilibrate NAI at 22°C for 15 min prior to use.
11 Add NAI to the RNA solution to reach a final concentration of 100 mM in a total volume of 20 µl.
12 Incubate the reaction mixture at 22°C for 5 min to allow selective modification.
13 Quench the reaction by adding 10 µl of 2 M DTT.
14 Purify the modified RNA using Micro Bio‐Spin Columns with Bio‐Gel P‐6, followed by ethanol precipitation.

#### In vivo NAI probing of RNA

15 Store the NAI probing buffer in an incubator overnight at 22°C.
16 Collect 5‐day‐old etiolated seedlings and treat them with 150 mM NAI at 22°C for 15 min to perform *in vivo* RNA modification.
17 Quench the reaction by adding a fivefold volume of DTT, followed by vigorous vortexing.
18 Flash‐freeze the treated seedlings in liquid nitrogen and grind them thoroughly into a fine powder.
19 Extract total RNA from the tissue obtained in the last step using the RNeasy Plant Mini Kit and perform two rounds of poly(A) RNA selection using the Poly(A) Purist Kit.

## Basic Protocol 2 CONSTRUCTION OF THE SHALiPE‐Seq LIBRARIES

This protocol outlines the step‐by‐step procedure for constructing SHALiPE‐Seq libraries, adapted from our previously established Structure‐Seq method (Ding et al., 2015). After NAI modification as described in Basic Protocol 1, the final guanine (G) in each G‐tract is preferentially modified, resulting in reverse transcription (RT) termination signals one nucleotide upstream of the modification site. For downstream PCR amplification, reverse transcription is performed using random hexamer primers (N6) that are appended with partial Illumina adapter sequences at their 5' ends. This random priming approach allows for transcriptome‐wide coverage by initiating RT at multiple locations within each transcript. Once the reverse transcription is completed, ssDNA ligation is carried out to attach the ssDNA linker to the 3’ end of the cDNA products. The library is then enriched via size selection and PCR amplification before being subjected to high‐throughput sequencing.

### Materials

RNase‐free TURBO DNase, 2 U/µl (Invitrogen, AM2238)
Poly(A)‐selected RNA (From Basic Protocol 1)
Nuclease‐free water (Invitrogen, AM9906)
RNA Clean & Concentrator Kit (Zymo, R1016)
dNTP set mix (100 mM each) (Invitrogen, 18427013)
Random Hexamer (CAGACGTGTGCTCTTCCGATCT(N)(N)(N)(N)(N)) fused to the Illumina TruSeq adapter
Superscript III Reverse Transcriptase (200 U/µl) (Invitrogen)
RT buffer, 5 × (See recipe)
RNaseOUT (40 U/µl) (Invitrogen, 10777019)
RNase H (10U/µl) (Invitrogen, AM2293)
Phenol:chloroform:isoamyl alcohol (25:24:1) (Sigma‐Aldrich, P2069)
Sodium acetate (pH 5.5) (3 M, Invitrogen, AM9740)
GlycoBlue (15 mg/ml) (Invitrogen, AM9515)
Ethanol
ssDNA linker (5’‐P‐NNNAGATCGGAAGAGCGTCGTGTAG‐3'‐C3)
Sodium chloride (5M) (Sigma‐Aldrich, S6546)
Circligase ssDNA Ligase (Epicentre, CL9021K)
Gel loading buffer II (Invitrogen, AM8547)
Novex TBE‐Urea Gels, 15% 10‐well (Invitrogen, EC6885BOX)
SYBR Gold (Invitrogen, S11494)
TEN250 buffer, 1 × (See recipe)
E.Z.N.A. gel extraction kit (Omega Bio‐tek, D2500‐01)
TopVision Agarose (Thermo Fisher Scientific, R0492)
Low‐molecular‐weight DNA ladder (New England Biolabs, N3233S)
Zero Blunt TOPO PCR Cloning Kit for Sequencing (Invitrogen, K2875J10)
KAPA Library Amplification Kits (Roche, KK2612)
NEBNext Multiplex Oligos for illumina (E7335S)

Agilent RNA 6000 Pico Kit (Agilent, 5067)

Microcentrifuge tubes, 1.7 ml
Eppendorf Centrifuge (5427R)
Benchtop contrifuge
NanoDrop One spectrophotometer (Thermo Fisher Scientific)
Illumina HiSeq 4000 platform
Vortexing machine
Rotary shaker (Eppendorf ThermoMixer)
Refrigerator
Thermal Cyclers for PCR
XCell SureLock Mini‐Cell Electrophoresis System (Thermo Fisher)
Benchmark Accuris MyView Compact UV Transilluminator (Merck)
Agarose Gel Electrophoresis Systems

#### DNase treatment

1 Prepare a 30‐µl DNase reaction on ice in a 1.7‐ml microcentrifuge tube by mixing 10 µl poly(A)‐selected RNA (0.2 µg/µl), 3 µl 10 × TURBO DNase buffer, 1 µl RNase‐free TURBO DNase (2 U/µl), and 16 µl RNase‐free water. Gently mix and incubate the reaction at 37°C for 30 min.
2 Add 3 µl resuspended DNase inactivation reagent (included in the RNase‐free TURBO DNase) to the reaction. Mix thoroughly and incubate at room temperature for 5 min.
3 Purify the DNase‐treated poly(A) RNA using the RNA Clean & Concentrator kit following the manufacturer's protocol.

#### cDNA preparation via reverse transcription

4 On ice, combine the DNase‐treated poly(A)‐selected RNA from step 3 with 1 µl of 10 mM dNTP mix and 1 µl random hexamer primer fused to the Illumina TruSeq adapter (10 µM), following the SuperScript III first‐strand synthesis system protocol.
5 Incubate the mixture at 65°C for 5 min to denature secondary structures, and then immediately place the tube on ice for at least 1 min.
6 On ice, prepare the cDNA synthesis mix according to the SuperScript III first‐strand synthesis system protocol, combining 2 µl home‐made RT buffer, 1 µl RNaseOUT (40 U/µl), and 1 µl SuperScript III Reverse Transcriptase (200 U/µl).
7 Mix the 10 µl cDNA synthesis mix prepared in step 6 with the 10 µl RNA/primer mixture from step 5. Briefly centrifuge the tube at less than 2000 rpm to collect the contents at the bottom using the benchtop centrifuge.
8 Incubate the combined mixture at 25°C for 10 min, and then continue incubation at 50°C for 50 min to complete the reverse transcription reaction.
9 Stop the reaction by heating the tube at 85°C for 5 min. Then immediately chill on ice and briefly centrifuge to collect the mixture.
10 Add 1 µl of RNase H to the tube to degrade residual RNA and incubate at 37°C for 20 min.
11 Purify the cDNA using phenol:chloroform *isoamyl alcohol* extraction.
  Use a 25:24:1 phenol:chloroform:isoamyl alcohol solution stored under a buffer layer. After mixing thoroughly, centrifuge 5 min at 16,000 × g, 4°C.
  CAUTION: Phenol, chloroform, and isoamyl alcohol are hazardous chemicals. Always wear appropriate personal protective equipment, including gloves and safety glasses.
12 Transfer the upper aqueous phase to a new 1.7‐ml microcentrifuge tube. Add an equal volume of chloroform, mix thoroughly, and centrifuge 5 min at 16,000 × *g*, 4°C.
13 To the upper aqueous phase transferred into a new 1.7‐ml microcentrifuge tube, add 1/10 volume of 3 M sodium acetate, 1 µl GlycoBlue (15 mg/ml), and 2.5 volumes of ethanol. Mix gently and incubate the mixture for at least 1 h on powdered dry ice or overnight at −80°C to precipitate the RNA.
  Store the RNA at −80°C to maintain stability.
14 Centrifuge the cDNA 20 min at 16,000 × *g*, at 4°C. Remove the supernatant, wash the pellet with 70% ice‐cold ethanol, and air‐dry briefly.

#### ssDNA ligation and cDNA size selection

15 Dissolve the cDNA pellet using 100 µM ssDNA linker on ice for 10 min. Then add 2 µl CircLigase 10 × reaction buffer, 1 µl 1 mM ATP, 1 µl 50 mM MnCl_2_, 1 µl CircLigase ssDNA ligase (100 U/µl), and RNase‐free water to the final volume. Incubate to ligate the first‐strand cDNAs.
16 Incubate the ligation reaction at 65°C for 12 h. Then, deactivate the CircLigase enzyme by heating the mixture at 85°C for 15 min.
17 Purify the ligated cDNA following the phenol:chloroform extraction procedure outlined in steps 11 to 13.
18 Centrifuge the purified ligated cDNA 20 min at 16,000 × *g*, at 4°C. Remove the supernatant, wash the pellet with 70% (v/v) ice‐cold ethanol, air‐dry briefly, and resuspend in 20 µl RNase‐free water.
19 Add 4 µl of gel loading buffer II to the ligated cDNA from step 18 and perform size selection of the ligated products on an 8.3 M urea‐10% (v/v) polyacrylamide gel. The gel is 0.75 mm thick, measuring 5.5 cm × 16.5 cm in size, with 1‐cm‐deep and 0.5‐cm‐wide wells. A lane containing 10‐bp DNA markers should be loaded.
  Perform a 30‐min pre‐run of the gel to raise the surface temperature to 55°C–60°C before loading samples.
20 Stain the gel with SYBR Gold and visualize the 10‐bp DNA markers and ligated cDNA bands under UV light. Excise the gel slice containing ligated cDNA fragments sized from above 60 nt up to the top of the well, thereby excluding the 52‐nt self‐ligated product of the reverse primer and ssDNA linker.
  The amount of cDNA product may be very low and is thus not detectable on the gel.
21 Transfer the excised gel slice into a 1.7‐ml microcentrifuge tube containing 1 × TEN250 buffer. Incubate at 4°C with constant rotary shaking for 2 h. Crushing the gel is unnecessary due to its thinness.
22 Purify the cDNA from the gel using the E.Z.N.A. gel extraction kit following the manufacturer's protocol.
23 Add to the eluted sample 1/10 volume of 3 M sodium acetate, 1 µl of GlycoBlue (15 mg/ml), and 2.5 volumes of ethanol. Mix well and incubate on powdered dry ice or at −80°C overnight (minimum 1 h) to precipitate the cDNA.
  The resulting cDNA can be stored overnight at −80°C for future use.
24 Centrifuge the size‐selected cDNA 20 min at 16,000 × *g*, 4°C. Remove the supernatant, wash the pellet with 70% ice‐cold ethanol, air‐dry briefly, and resuspend in 10 µl RNase‐free water.

#### PCR cycle optimization and PCR pool amplification

25 Set up a PCR cycle optimization reaction by mixing the following in a 25 µl total volume including 1 µl of the size‐selected cDNA from step 24: 12.5 µl of KAPA Library Amplification Ready HIFI mix, 1 µl of NEBNext Universal Primer for Illumina (5 µM), and 1 µl of NEBNext indexed primer for Illumina  (5 µM). In addition, prepare a primer–dimer control reaction by excluding the size‐selected cDNA from the mix.
  Other high‐fidelity polymerases with similar characteristics may be suitable for this protocol; however, their performance has not been tested.
26 Perform the PCR amplification under the following conditions and remove 4 µl of the reaction at cycle numbers 10, 20, 25, 30, and 35.

**Table:** 

Cycle number	Denature	Anneal	Extend
Initial denaturation	95°C, 3 min		
1–35	98°C, 20 s	60°C, 15 s	72°C, 30 min
Final extension			72°C, 5 min
27 Run all samples on a 1.5% (w/v) agarose gel and visualize the DNA bands using SYBR Gold staining.
  Typically, a smear appears after 25 cycles of amplification, whereas nonspecific bands in the primer–dimer control may become visible around 35 cycles.
28 Perform PCR amplification using the remaining 9 µl of ligated cDNA from step 25. Use the same reaction conditions as described in step 26 and apply the optimal number of PCR cycles determined in step 27.
  The PCR products can be stored at −20°C overnight or for several months without significant degradation.

#### Size selection of PCR pool amplification products

29 Add 5 µl of 6 × gel loading buffer to the PCR products obtained in step 28 and load the mixture on a 1.5% (w/v) agarose gel. Include a 1‐kb plus DNA ladder as size markers.
30 Visualize the gel under UV illumination after staining with SYBR Gold. Cut out the gel region corresponding to fragments between 150 bp and 650 bp to eliminate primer–dimer bands (∼130 bp). Transfer the excised gel slice (approximately 0.3 g) into a 1.7‐ml microcentrifuge tube.
31 Extract DNA from the gel slice using a gel extraction kit. We use the E.Z.N.A. gel extraction kit, following the manufacturer's instructions. Other kits may also be suitable, although they have not been tested in this protocol.
  The extracted PCR products can be stored at −20°C overnight or for several months for future use.
32 Perform the gel extraction process two additional times.
  Performing the agarose gel purification three times ensures that the final sample contains less than 10% primer–dimer contamination, significantly improving the yield of usable reads for deep sequencing.

#### Library validation and deep sequencing

33 Use the Zero Blunt TOPO PCR Cloning Kit to subclone the purified PCR products following the protocols provided by the manufacturer.
34 Perform DNA extraction from 48 colonies and conduct sequencing via standard capillary sequencing.
35 Conduct BLAST analysis of sequencing results against the transcriptome and genome of the organism under study to identify the sequence. Verify the specificity of NAI modification, which should terminate transcripts 1 nt before the RT stalling, and identify the number of colonies containing primer–dimer sequences. Ensure that the primer–dimer sequence occurs at a low frequency (ideally 5 out of 48 colonies).
  Ensure that the number of colonies with primer–dimer sequences is less than 5 out of 48 samples.
36 Determine the library concentration for deep sequencing by using NanoDrop.
37 Perform high‐throughput sequencing of each strand‐specific library obtained in step 32 using an Illumina HiSeq 4000 platform, following the manufacturer's protocols.

## Basic Protocol 3 MEASUREMENT OF THE RNA G‐QUADRUPLEX FOLDING STATUS BASED ON SHALiPE‐Seq LIBRARIES

**Figure 2:**
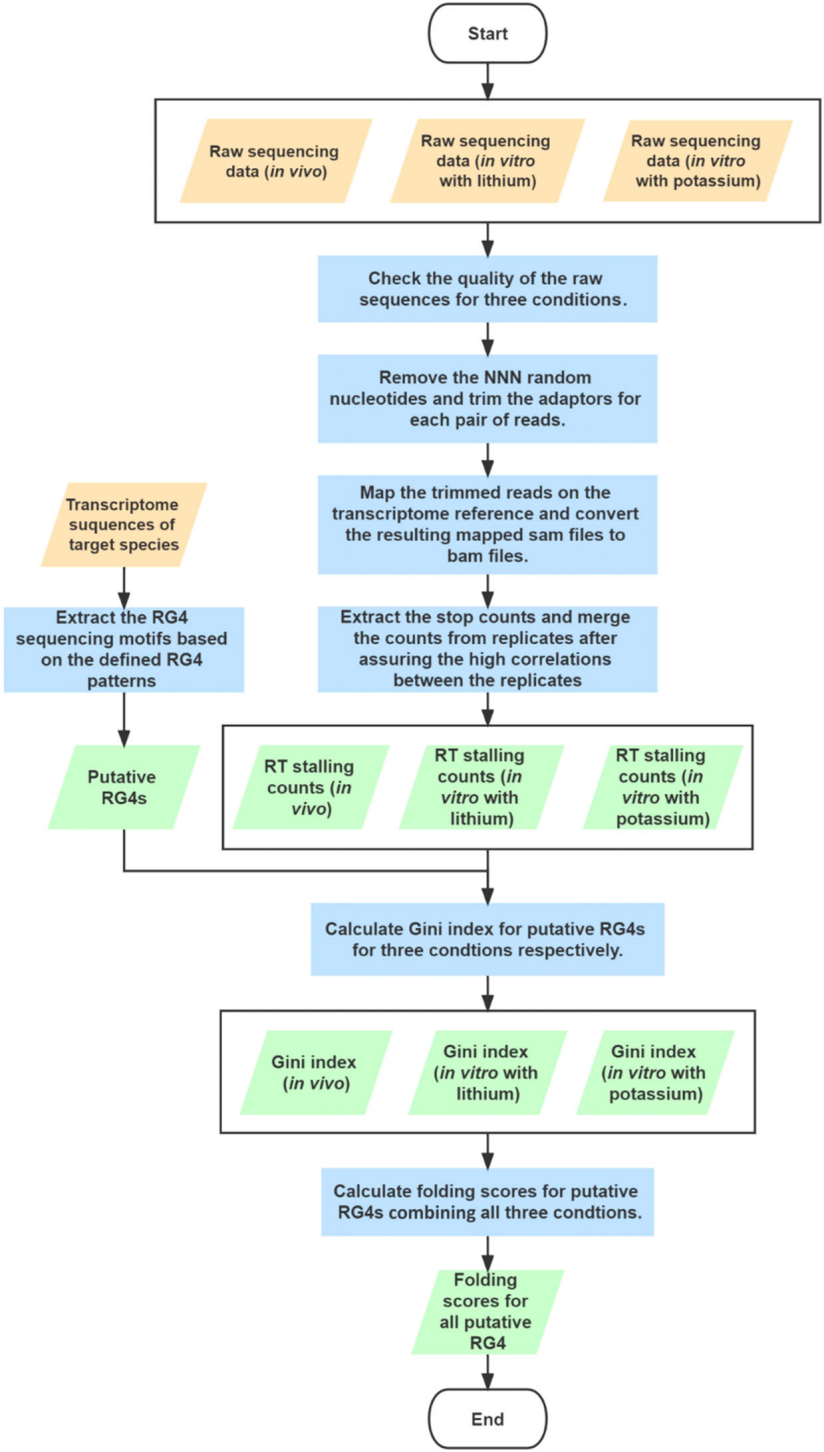
Overview of the data analysis pipeline described in this article.

This section describes the computational pipeline used to assess the folding status of RNA G‐quadruplexes (RG4s) from high‐throughput sequencing data. For each biological replicate under each experimental condition, RT stalling counts were calculated based on the mapped reads. Replicates displaying high correlation within the same condition were merged to improve signal robustness. Following the integration of RT stalling profiles, Gini index values were computed for each condition to quantify the signal distribution and assess structural uniformity. Using these values, *in* *vivo* folding scores were generated for each putative RG4 motif, as defined by established sequence criteria specific to RG4 formation. These scores reflect the likelihood of each RG4 motif forming a stable structure *in vivo*. An overview of the data analysis workflow is illustrated in Figure 2.

### Materials

Program FastQC v.0.11.9 (https://github.com/s‐andrews/FastQC)
Program Trimmomatic v.0.39 (http://www.usadellab.org/cms/index.php?page=trimmomatic)
Program Bowtie v.1.0.1 (https://bowtie‐bio.sourceforge.net/index.shtml)
Program Samtools v.1.4.1 (https://www.htslib.org/)
Python package HTseq v.0.7.2 (https://htseq.readthedocs.io/en/latest/)
Python package pysam v.0.11.2.2 (https://github.com/pysam‐developers/pysam)

#### Sequencing read mapping

1 The quality of raw sequencing reads was assessed using computational tools. Only reads passing quality control were subjected to downstream analyses.
  We used FastQC version 0.11.9 in our previous study.
2 The NNN random nucleotides from the single‐stranded DNA (ssDNA) linker were trimmed from the 5′ end of each read pair. Adapters were removed from the reads.
  We used Trimmomatic version 0.39 in our previous study.
3 Map the trimmed reads on the transcriptome reference (for *Arabidopsis thaliana*, we used transcriptome version TAIR10). Use Samtools‐1.4.1 to convert the SAM files (mapping result) to BAM files and index them.
  Different software can be used for mapping reads. We employed Bowtie 1.0.1 for mapping reads, with a minimum read length requirement of 21 nt. This read length was deemed appropriate for achieving unique mapping in Arabidopsis thaliana.
  To account for potential errors during PCR and sequencing, we permitted up to three mismatches in the mapped reads without any insertions or deletions. Further processing was carried out using only reads that were uniquely mapped.
4 RT stop counts were extracted. For each mapped read, the reverse transcription stop site was defined as the nucleotide located one nucleotide upstream of the 5′ end of the aligned read.
  We used modules from two Python packages: HTseq version 0.7.2 and pysam version 0.11.2.2. During read mapping to the reference, the stop position of the actual structure corresponds to the nucleotide that is located 1 nt upstream of the 5' end of the mapped read.
  If the correlation is low between the replicates, the data cannot be subjected to further steps.

#### Gini index and folding score calculation

5 To identify putative RNA G‐quadruplex (RG4) sequences within the transcriptome, we used a motif‐scanning approach based on known structural subclasses of RG4s. The motifs were extracted using regular expressions designed to capture distinct classes of RG4 structural patterns, as follows:
  (1) Canonical G_3_‐RG4s with four tracts of three consecutive guanines (G_3_), separated by loops of 1–15 nucleotides. The pattern is [G_3_N_1‐15_G_3_N_1‐15_G_3_N_1‐15_G_3_].
  (2) Noncanonical G_2_‐RG4s with four tracts of two consecutive guanines (G_2_), separated by loops of 1–9 nucleotides. The pattern is [G_2_N_1‐9_G_2_N_1‐9_G_2_N_1‐9_G_2_].
  (3) G_3_‐RG4s having a vacant G‐quartet layer with three G_3_ tracts and one G_2_ tract, allowing a gap in one G‐quartet layer. Loop lengths range from 1–9 nucleotides. The patterns are [G_3_N_1‐9_G_3_N_1‐9_G_3_N_1‐9_G_2_] or [G_2_N_1‐9_G_3_N_1‐9_G_3_N_1‐9_G_3_].
  (4) G_3_‐RG4s having bulged loops with G_3_, with loop length ranging from 1 to 9 nt. The pattern is [G_3_N_1‐9_(G_2_HGN_1‐9_ or GHG_2_N_1‐9_) (G_2_HGN_1‐9_ or GHG_2_N_1‐9_) G_3_].
  In the definitions, N represents any of the four nucleotides A, C, G, or U, while H represents any of the nucleotides A, C, or U.
  The definition of the patterns of RG4 can be different in diverse species. Some high G‐content species might have RG4 motifs with more than three layers of G‐tracts.
6 To quantify the folding status of each putative RG4 motif identified in step 5, we calculated the Gini index based on the SHALiPE‐Seq read counts mapped to G residues within the G‐tracts of each motif. The Gini index provides a measure of the signal distribution unevenness, reflecting the degree of reverse transcriptase stalling at specific G positions.
$$\mathrm{Gini}\;=\frac{\Sigma_{i=1}^{n}\Sigma_{j=1}^{n}\left|ri-rj\right|}{2n^{2}\bar{r}}$$
  Here, n represents the number of G residues in the G‐tracts (continuous runs of guanine in the G‐rich region), *r_i_* represents the RT stop read counts in SHALiPE profiles at position *i*, and $\bar{r}$ represents the average RT stop read counts of all G residues.
  A higher Gini index suggests strong RT stalling at specific G residues, consistent with folded RG4 structures. A lower Gini index indicates a more uniform signal, suggesting unfolded or weakly folded regions.
7 To determine the *in vivo* folding status of individual RG4 motifs, we calculated an *in vivo* folding score by comparing SHALiPE‐Seq Gini indices between *in vivo* and *in vitro* conditions using the following equation.
$$\mathrm{folding}\;\mathrm{score}\;=\frac{\mathrm{Gini}\left(\;\mathrm{in}\;\mathrm{vivo}\;\right)-\;\mathrm{Gini}\left(\;\mathrm{in}\;\mathrm{vitro}\;Li^{+}\right)}{\mathrm{Gini}\left(\;\mathrm{in}\;\mathrm{vitro}\;K^{+}\right)\;-\;\mathrm{Gini}\left(\;\mathrm{in}\;\mathrm{vitro}\;Li^{+}\right)}$$
  Only RG4 regions meeting both of the following criteria were included in this analysis: Gini (*in vitro* K^+^)/Gini (*in vitro* Li^+^) ≥ 1.1, ensuring the selected regions exhibit potassium‐dependent folding *in vitro*; the average SHALiPE‐Seq read count (RT‐stop site coverage) at G residues must be higher than 50 reads/nt.
  Gini (in vitro K^+^) is required to be higher than Gini (in vitro Li^+^) as more rG4s tend to form in the K^+^ condition than in the Li^+^ condition. The minimum average read count (50) can be altered in terms of the transcriptome size and the sequencing depth.

## Reagents and Solutions

### 1 × TEN250 buffer

10 mM Tris (pH 7.5) (Sigma, T2319)
1 mM EDTA (Invitrogen, 03690)
250 mM NaCl (Invitrogen, AM9759)

### 5 × RT buffer

20 mM Tris·HCl (pH 8.3) (Sigma, T2319)
100 mM LiCl (Sigma, L7026)
3 mM MgCl_2_ (Sigma, 63020)
1 mM DTT (Thermo, R0862)
Store at −20°C for up to 1 year.

### 6 × gel loading buffer

4 g sucrose
25 mg bromophenol blue
Bring volume to 10 ml with nuclease‐free water; the solution can be stored up to 1 year at room temperature.

## Commentary

### Background Information

Earlier strategies for detecting RNA G‐quadruplexes (RG4s) in fixed or live cells employed G‐quadruplex‐specific antibodies (Biffi et al., 2014), small‐molecule ligands (Laguerre et al., 2015; Renard et al., 2019; Yang et al., 2018), and fluorescent probes (Chen et al., 2016, 2018). While these tools have facilitated RG4 identification, they fall short in reliably assessing RG4 folding status *in vivo* due to several key limitations: 1) The antibodies/ligands/probes are capable of inducing RNA G‐quadruplex folding. The folding is likely to be triggered during experimental procedures rather than reflecting the native folding status in the living cells (Guo & Bartel, 2016; Kharel et al., 2020; Yang et al., 2020). 2) It is not possible to determine the RG4 formation of individual G‐rich regions of interest within living cells. 3) Excluding potential RG4 folding in fixing, permeabilizing, or staining cells was not feasible (Guo & Bartel, 2016).

To overcome the aforementioned limitations, two cell‐permeable chemicals have been employed in the development of RG4‐specific structure‐probing techniques. The first chemical is 2‐methylnicotinic acid imidazolide (NAI), which preferentially modifies each G‐tract's last G residue. It is likely due to the exposure of the 2′‐hydroxyl of the last G residues of the folded RG4 (Guo & Bartel, 2016; Kwok et al., 2016b; Yang et al., 2020). In addition to NAI, other chemicals that can modify 2′‐hydroxyl can also be used to probe RG4s in theory, including 2A3 (2‐aminopyridine‐3‐carboxylic acid imidazolide), 1‐methyl‐7‐nitroisatoic anhydride (1M7), and others. This special modification pattern is subsequently identified using reverse transcription (RT) stalling. The other chemical is dimethyl sulfate (DMS). The N^7^ position of guanine (N^7^G) can be modified by DMS with a high concentration, which can block the capability of RG4 folding in the presence of K^+^ *in vitro* when the RG4 sequence is not folded (Guo & Bartel, 2016). If RG4 is folded *in vivo*, the DMS cannot modify the N^7^G. Thus, RG4 can be folded in the presence of K^+^ *in vitro*, which could be determined by RT stalling (Guo & Bartel, 2016). The folding status of RG4 *in vivo* can be determined indirectly. However, the high concentration of DMS is extremely toxic to living cells (Wang et al., 2019). Our new method, SHALiPE‐Seq, takes advantage of the property of NAI to map RG4 folding states *in vivo*.

Our SHALiPE‐Seq method combines chemical structure probing with high‐throughput sequencing to quantitatively assess the transcriptome‐wide folding status of RNA G‐quadruplexes (RG4s). This approach offers two key advantages: 1) It is the first technique capable of directly and quantitatively evaluating RG4 folding within living cells. 2) It enables the comprehensive identification of RG4s across an entire transcriptome in a single experiment. Using SHALiPE‐Seq, our previous work demonstrated that RG4s are indeed folded *in vivo* in both *Arabidopsis thaliana* and rice, providing direct evidence for the existence of these structures in living eukaryotic cells (Yang et al., 2020). Given that NAI is compatible with a broad range of organisms, including human cells, *Drosophila*, and wheat, SHALiPE‐Seq can be readily adapted for RG4 studies in diverse species.

### Critical Parameters

#### RNA handling during RNA folding and probing

Precautions should be taken when working with RNA. Unintentional RNA degradation resulting from RNase contamination is a pervasive problem, and the repercussions are especially severe during denaturation and renaturation.

#### K^+^, Li^+^, and NAI concentration optimization

*In vitro* probing under K⁺ and Li⁺ conditions served as benchmarks for folded and unfolded RG4 states, respectively. Thus, precise concentrations of K⁺ and Li⁺ are critical for accurately assessing RG4 folding. Biophysical techniques, such as circular dichroism (CD) spectroscopy, can be used to optimize K⁺ concentrations by measuring ellipticity at 262 nm, where increased signals with rising K⁺ levels indicate stronger RG4 folding. The concentration of NAI is also crucial, as its modification efficiency can vary across species and experimental conditions such as temperature. To enable extensive modification while maintaining single‐hit kinetics, the NAI concentration should be optimized accordingly. In our *in vivo* experiments, we used 150 mM NAI, but we recommend empirically determining the optimal concentration when applying SHALiPE‐Seq to different organisms or conditions.

#### Reverse transcription

Reverse transcription is the key step in SHALiPE‐Seq. The thermostable SuperScript III reverse transcriptase is used in this method to facilitate the reverse transcription conducted at relatively high temperatures to avoid the formation of RNA structures during the reaction. Other reverse transcriptases with comparable properties can also be used.

#### PCR cycle optimization

During PCR cycle optimization, the objective is to use the minimal number of cycles necessary to amplify the library while reducing read duplication. If the optimal cycle number is too high, consider increasing the amount of cDNA input in the PCR reaction to achieve sufficient amplification with fewer cycles.

### Data analysis

In the data analysis pipeline, RT‐stop site coverage is a critical parameter for confidently assessing RG4 folding status. In our previous studies, G residues with an average read count (RT‐stop site coverage) ≥50 reads/nt were included in the calculation of Gini indices and folding scores. This threshold ensures data reliability but may be adjusted, particularly lowered, when strong and clear modification patterns are observed. Additionally, to establish reliable benchmarks for folded and unfolded states, the Gini index under in vitro K⁺ conditions must exceed that under in vitro Li⁺ conditions.

### Troubleshooting

Table 1 provides troubleshooting guidance.

**Table 1:** Troubleshooting Guide for SHALiPE‐seq

Problem	Possible cause	Solution
Gini index too high even under the Li^+^ condition	1) These RG4s might be strongly folded. 2) It is also possible that the potassium level is high during RNA extraction.	1) These individual RG4s are subjected to further biophysical analysis, such as circular dichroism spectroscopy to determine the folding capacity of these RG4s. 2) The RNA samples can first be dialyzed against Li^+^. Li^+^ is used to replace Na^+^ or K^+^ that might be introduced during the RNA extraction process.
Low ssDNA ligation efficiency	The ligation efficiency might be affected by the potential ssDNA secondary structure.	The reaction temperature can be elevated to 65°C, which is higher than the manufacturer‐recommended 60°C, and the reaction time can be extended to 12 h.
Low mapping rate	1) Since the reverse primer can ligate with the ssDNA linker, there is a large proportion of this type of self‐ligated product that might be sequenced at the same time. 2) The raw reads were not properly processed by trimming the adapters and removing the random nucleotides at the ssDNA linker.	1) Clean‐cut the self‐ligated products during the cDNA size selection. 2) Follow the protocol to process the raw data.
Less RT stalling signals	1) A low NAI concentration might cause insufficient modification, leading to less RT stalling. 2) A high NAI concentration might cause over‐modification, which leads to extensive RT stalling enriched close to the reverse transcription starting sites. These short RT products are likely to be extremely short and removed during library construction.	Test the NAI concentrations before starting SHALiPE‐Seq. To maintain single‐hit kinetics while enabling extensive modification, the NAI concentration should be optimized accordingly.

### Understanding Results

Our SHALiPE‐Seq technique generates three distinct Gini index datasets for each predicted RG4 across the transcriptome, reflecting three structural states: unfolded *in vitro*, folded *in vitro*, and folded *in vivo*. The calculated *in vivo* folding score, ranging from 0 to 1, serves as an indicator of the folding status. Values near 0 suggest an unfolded state, whereas scores approaching 1 indicate that the RG4 is likely folded. Each candidate RG4 is assigned an individual *in vivo* folding score based on SHALiPE‐Seq profiling. When applied to *Arabidopsis*, the *in vivo* folding score distribution was skewed toward higher values, with a median of 0.755, implying widespread RG4 folding in this species. In contrast, rice exhibited an even higher median score of 0.938, indicating a greater prevalence and stability of RG4 folding in its transcriptome compared to *Arabidopsis*. Collectively, these findings demonstrate that RNA G‐quadruplexes tend to be predominantly folded within plant transcriptomes.

### Time Considerations

The construction of SHALiPE libraries ready for deep sequencing can be completed within 14–17 days, starting from chemical synthesis to chemical treatment. Normally, we could handle 8 samples at most. On day 1, the SHAPE NAI chemical is synthesized and stored. On day 2, plants are treated with the NAI chemical followed by RNA extraction. On day 3, two rounds of poly‐A selections can be done to enrich mRNAs. On day 4, *in vitro* RNA samples are folded in Li^+^/K^+^ conditions, followed by the probing with NAI. On day 5, the RT reactions will be performed. On day 6, the ssDNA adaptor ligation will be performed. On day 7, the ligated ssDNA will be subject to size selection. PCR cycle optimization and the size selection of dsDNA can be completed in 2 to 5 days. Library validation usually takes ∼5 days. However, the protocols included flexible stopping points to allow for adjustments as needed. After obtaining the deep sequencing data, the data analysis will take about 10 days from the read mapping to the RG4 folding scores, which depends highly on the performance of the computer hardware.

### Author Contributions

**Bibo Yang**: Conceptualization; data curation; methodology; visualization; writing original draft; writing review and editing. **Yiliang Ding**: Conceptualization; funding acquisition; supervision; writing review and editing. **Yueying Zhang**: Conceptualization; supervision; writing review and editing.

### Conflict of Interest

The authors declare no conflict of interest.

## Data Availability

The data that support the findings of this study are available from the corresponding author upon reasonable request.
